# 

**Published:** 1891-01

**Authors:** 


### CONTENTS FOR JANUARY, 1891.

ORIGINAL COMMUNICATIONS.

Three Cases of Tubercular Peritonitis Operated on by Abdominal Section During the Year. By JAMES F. W. ROSS, M. D. .... 321
The Renal Status of Opium Habitués. By J. B. MATTISON, M. D. .... 332
Neurasthenia and Nasal Disease. By FRANK HAMILTON POTTER, M. D. .... 337

TRANSLATIONS.

Koch's Tuberculosis Cure .... 345

SOCIETY PROCEEDINGS.

Buffalo Pathological Society .... 350

PROGRESS IN MEDICAL SCIENCE.

Medicine .... 355
Physiological Chemistry .... 356

SELECTIONS.

Notes Upon Somnal, The New Hypnotic .... 359
Therapeutic Notes and Miscellany .... 362

EDITORIAL.

The Heating of the Street Cars .... 365
University of the State of New York .... 366
The Erie County Eye, Ear, and Throat Infirmary .... 368
*The University Medical Magazine.* Professor Reise's Letters .... 369

PERSONAL.

Dr. Gustav A. Pohl .... 370
Col. Charles Sutherland—Dr. Carl Weidner—Dr. L. Bradley Dorr—Dr. J. C. Sexton—Dr. T. Haven Ross .... 370

OBITUARY.

Surgeon-General Baxter .... 371
Dr. Sidney Allan Fox .... 371
Dr. James L. Stewart—Dr. William H. McReynolds .... 372

SOCIETY MEETINGS.

Seventh International Congress of Hygiene and Demography .... 372
The Medical Society of the State of New York—The Medical Union—The District Medical Society .... 373

JOURNALISTIC NOTES.

*The Bacteriological World* .... 373

BOOK REVIEWS.

Epilepsy .... 374
Medical Diagnosis with Special Reference to Practical Medicine .... 375
A Treatise on the Diseases of Infancy and Childhood .... 376
Wood's Medical and Surgical Monographs—Saunders' Qestion Compends, No. 15 .... 377
Cyclopedia of the Diseases of Children.—The Physician's All-Requisite Time and Labor-Saving Account Book .... 378
Index-Catalogue Surgeon-General's Office—The Patient's Record—Bacteriological Technology for Physicians—The Medical Bulletin Visiting List .... 379
The Physician's Visiting List (Blakiston's) .... 380
Books and Pamphlets Received .... 380
Miscellany .... 382

